# Above- and Belowground Biomass Allocation in Shrub Biomes across the Northeast Tibetan Plateau

**DOI:** 10.1371/journal.pone.0154251

**Published:** 2016-04-27

**Authors:** Xiuqing Nie, Yuanhe Yang, Lucun Yang, Guoying Zhou

**Affiliations:** 1 Northwest Institute of Plateau Biology, Chinese Academy of Science, Xining, 810008, China; 2 Key Laboratory of Tibetan Medicine Research, Chinese Academy of Science, Xining 810008, China; 3 State Key Laboratory of Vegetation and Environmental Change, Institute of Botany, Chinese Academy of Sciences, Beijing, 100093, China; 4 University of Chinese Academy of Science, Beijing, 100049, China; Chinese Academy of Forestry, CHINA

## Abstract

Biomass partitioning has been explored across various biomes. However, the strategies of allocation in plants still remain contentious. This study investigated allocation patterns of above- and belowground biomass at the community level, using biomass survey from the Tibetan Plateau. We explored above- and belowground biomass by conducting three consecutive sampling campaigns across shrub biomes on the northeast Tibetan Plateau during 2011–2013. We then documented the above-ground biomass (AGB), below-ground biomass (BGB) and root: shoot ratio (R/S) and the relationships between R/S and environment factors using data from 201 plots surveyed from 67 sites. We further examined relationships between above-ground and below-ground biomass across various shrub types. Our results indicated that the median values of AGB, BGB, and R/S in Tibetan shrub were 1102.55, 874.91 g m^-2^, and 0.85, respectively. R/S showed significant trend with mean annual precipitation (MAP), while decreased with mean annual temperature (MAT). Reduced major axis analysis indicated that the slope of the log-log relationship between above- and belowground biomass revealed a significant difference from 1.0 over space, supporting the optimal hypothesis. Interestingly, the slopes of the allometric relationship between log AGB and log BGB differed significantly between alpine and desert shrub. Our findings supported the optimal theory of above- and belowground biomass partitioning in Tibetan shrub, while the isometric hypothesis for alpine shrub at the community level.

## Introduction

Root: shoot ratio (R/S) is one of the most common descriptors of the relationship between root and shoot biomass, which has become a key method for estimating below-ground biomass (BGB) from above-ground biomass (AGB). The partitioning of above- and belowground biomass is a core parameter of carbon cycling in terrestrial biomes [1,2]. From a physiological perspective, R/S has been interpreted as a critical parameter as reflecting the differential investment of photosynthates between the aboveground and belowground organs [3,4]. Therefore, quantifying this ratio and its relationships with climatic factors can not only improve the accuracy of root biomass estimates, but also be important for mechanistic understanding of terrestrial carbon cycles [2–5].

Biomass partitioning is usually explained by isometric and optimal hypotheses [6–8]. The isometric allocation hypothesis suggests that the slope of the log-log relationship between above AGB and BGB is not significantly different from 1.0 and does not show any significant change with environment conditions [9,10]. Compared with this hypothesis, the optimal hypothesis indicates that plants respond to variation in environment conditions by allocation biomass among various organs to capture nutrients, water, and light to maximize their growth rate [9,11,12]. Biomass partitioning has been widely examined across various biomes [2–5,9, 13–18]. However, the strategies of allocation in plants still remain contentious [10,14–19]. For grasslands, the biomass partitioning relationship of AGB and BGB fits isometric hypothesis in community type from Tibetan Plateau to China [8,9], while there are also evidences that reflect the allometric biomass partitioning relationship on the Tibetan Plateau and patterns of biomass allocation in China’s grasslands also do not fit the isometric hypothesis from individual- level observations [16]. In forest ecosystems, evidences from temporal observations demonstrate the isometric biomass partitioning hypothesis [18]. Nevertheless, optimal partitioning patterns exist both intraspecifically and interspecifically for arboreal conifers [8]. Previous studies shed light on how plats adjust C allocation across various biomes, yet, it is less involved in shrubs. Some previous studies assessed biomass allocation of shrubs are mainly focused on individual levels [20–22], and are not only proven the isometric biomass partitioning relationship [20,21] but also exist the allometry hypothesis [20,22], and they are less to research at communities level. Furthermore, allocation relationships between log AGB and log BGB is unknown in the northeast Tibetan Plateau shrub biomes.

In this study, we evaluated AGB and BGB allocation in the Tibetan Plateau shrub biomes. To this end, we conducted a regional sampling survey during the 2011–2013 and sampled 201 sites across the northeast Tibetan Plateau. We then determined AGB and BGB for those samples. Using these datasets, we examined relationships between R/S and climatic factors, and also explored the hypothesis of biomass allocation. Overall, this study aimed to answer the following questions: (1) How climatic factors effect biomass allocation? (2) What is the relationship between AGB and BGB in shrub biomes across the northeast Tibetan Plateau?

## Materials and Methods

### Study area

The study area covered with shrubs on the northeast Tibetan Plateau, extending form latitudes of 31°52′33.92″to 38°05′09.15″ N and longitudes of 94°30′38.50″to 102°22′33.94″ E (S1 Table). Shrubs are one of the most important biomes in study area [23] and dominantly consisted of woody plants in ecosystem, with the average of height below 5m, and the coverage more than both 30% and 40% [24]. Alpine shrubs and desert shrubs are predominant biomes in shrub ecosystem across northeast Tibetan Plateau [23]. Alpine shrubs occur at the wet regions and can endure cold and semiarid environments, such as *Potentilla fruticosa* Linn, *Rhododendron thymifolium* Maxim, *Sibiraea laevigata* (Linn) Maxim and *Rhododendron capitatum* Maxim biomes, while desert shrubs are distributed at drier areas and prevailingly consist of plants can bear severe drought, such as *Nitraria tangutorum* Bobr, *Kalidium foliatum* (Pall.) Moq, *Salsola abrotanoides* Bunge and *Sympegma ragelii* Bunge biomes [23]. The mean annual temperature and mean annual precipitation of the study area range from -5.6 to 8.9°C and from 17.6 to764.4 mm, separately [25].

### Field biomass survey

We systematically selected 201 plots surveyed from 67 sites across the northeast Tibetan Plateau during the summers (July to August) from 2011–2013. In each site, no specific permits were demanded for collecting samples and the field studies did not involve endangered or protected species. The plots were 5 m × 5 m for alpine shrub and 10 m × 10 m for desert shrub. There are three plots in each site and the distance of two plots was between 5 and 50 m. In each plot, we set a subplot (1 m × 1 m), and all shrub plants in three subplots (1 m × 1 m) were harvested to measure AGB [26]. Each corresponding subplot was excavated until the maximum root depth, which were sampled to determine BGB. Root samples were soaked in water. Dead roots were removed [14] and live roots were distinguished by their color, consistency and attached fine roots [27]. Live shoot and root biomass were used oven-dried at 65°C to constant weight, and weighed to the nearest 0.01 g, and used to evaluate biomass partitioning patterns and their relationships with climatic variables [4,5].

### Data analysis

First, due to their log-normal distributions of raw biomass data, we calculated the median values of AGB, BGB, and R/S for all sampling sites. We then classified all sites from alpine shrub to desert shrub. We calculated overall AGB, BGB, and R/S for the two types of shrub biomes on the Tibetan Plateau.

Second, to investigate the potential effects of climatic factors on R/S, mean annual temperature (MAT) and mean annual precipitation (MAP) were extracted for each site from the worldclim database (http://www.worldclim.org/) with a spatial resolution of 1 × 1 km^2^ [28–30].

Third, the relationship between log-transformed above- and belowground biomass was explored by Ordinary least squares (OLS) analyses and reduced major axis (RMA) analyses [31,32]. The slope (a) and *y*-intercept (logβ) of log–log linear functions for RMA were determined by the software package ‘Standardized Major Axis Tests and Routines’ [33].

## Results

### Size of AGB, BGB and R/S

**Fig 1:**
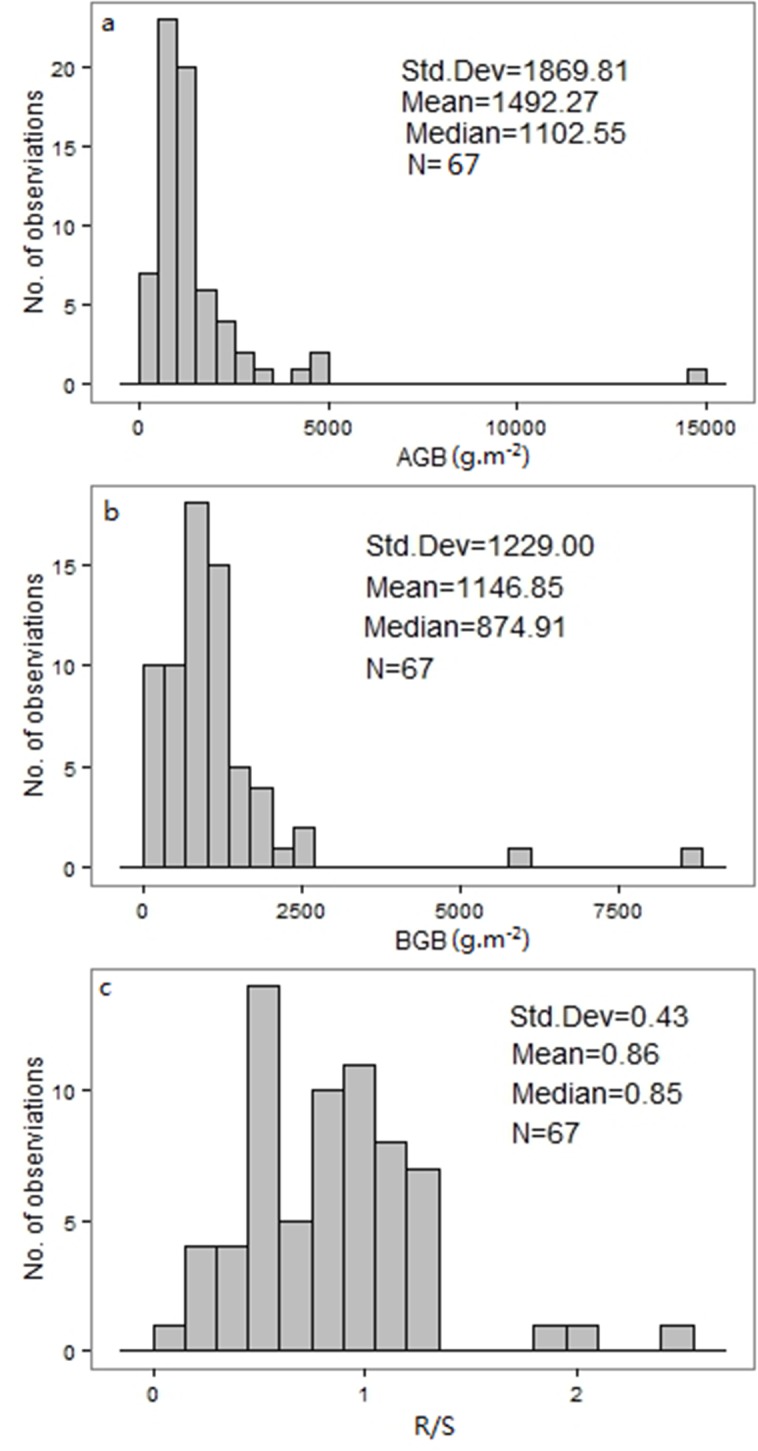
**Frequency distributions of (a) above-ground biomass (AGB), (b) below-ground biomass (BGB), and (c) root: shoot ratio (R/S) in shrub.** Their mean values, median values, Std. Dev and N are presented.

AGB, BGB and R/S exhibited large variations across all the sites, ranging from 340.34 to 14623.12 g m^-2^ for AGB, while BGB ranged from 89.36 to 8565.27 g m^-2^ and R/S ranged from 0.13 to 2.51 (Fig 1). The median values of AGB for alpine shrub and desert shrub were 1036.36 and 1194.36 g m^-2^ separately. The median values of BGB for alpine shrub and desert shrub were 951.55 and 603.36 g m^-2^. And the median values of R/S of alpine shrub and desert shrub were 0.95 and 0.44 (Table 1). The mean values of AGB, BGB, and R/S for shrub (alpine shrub and desert shrub) were 1492.27, 1146.85 g m^-2^ and 0.86, respectively (Fig 1).

**Table 1:** The median values of above-ground biomass (AGB), below-ground biomass (BGB) and root: shoot ratio (R/S) for various shrub types on the Tibetan Plateau.

Shrub types	AGB (g m^-2^)		BGB (g m^-2^)		R/S		n
	Median	Range	Median	Range	Median	Range	
Alpine shrub	1036.36	340.34–4816.52	951.55	170.21–2597.28	0.95	0.45–2.02	49
Desert shrub	1194.36	426.03–14623.12	603.36	89.36–8565.27	0.44	0.13–2.51	18
Overall	1102.55	340.43–14623.12	874.91	89.36–8565.27	0.86	0.13–2.51	67

### Effects of MAT, MAP on R/S

**Fig 2:**
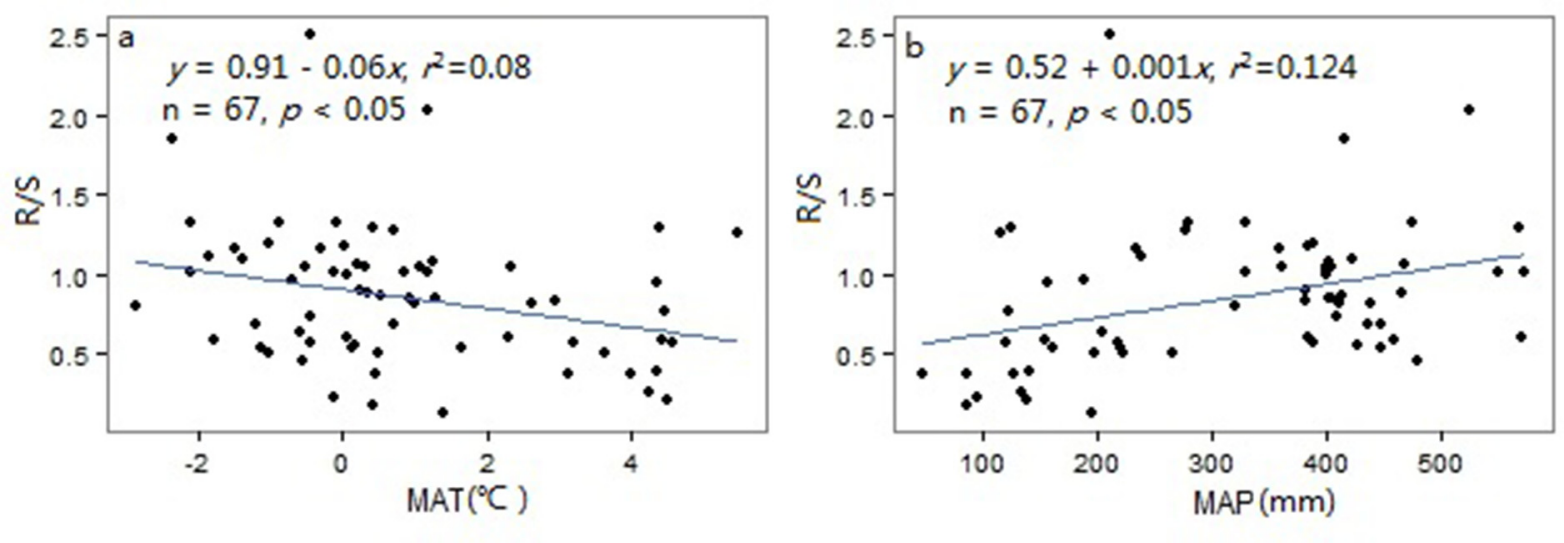
Relationships of root: shoot ratio (R/S) with climatic factors for shrub biomes on the Tibetan Plateau. (a, b) R/S vs. MAT, mean annual temperature, MAP, mean annual precipitation.

The R/S in Tibetan shrubs dropped significantly with increasing in MAT (*r*^*2*^ = 0.08, *p* < 0.05 Fig 2A), while showed significantly positive trend with MAP (*r*^*2*^ = 0.12, *p* < 0.05 Fig 2B).

### Allometric relationships between AGB and BGB

**Fig 3:**
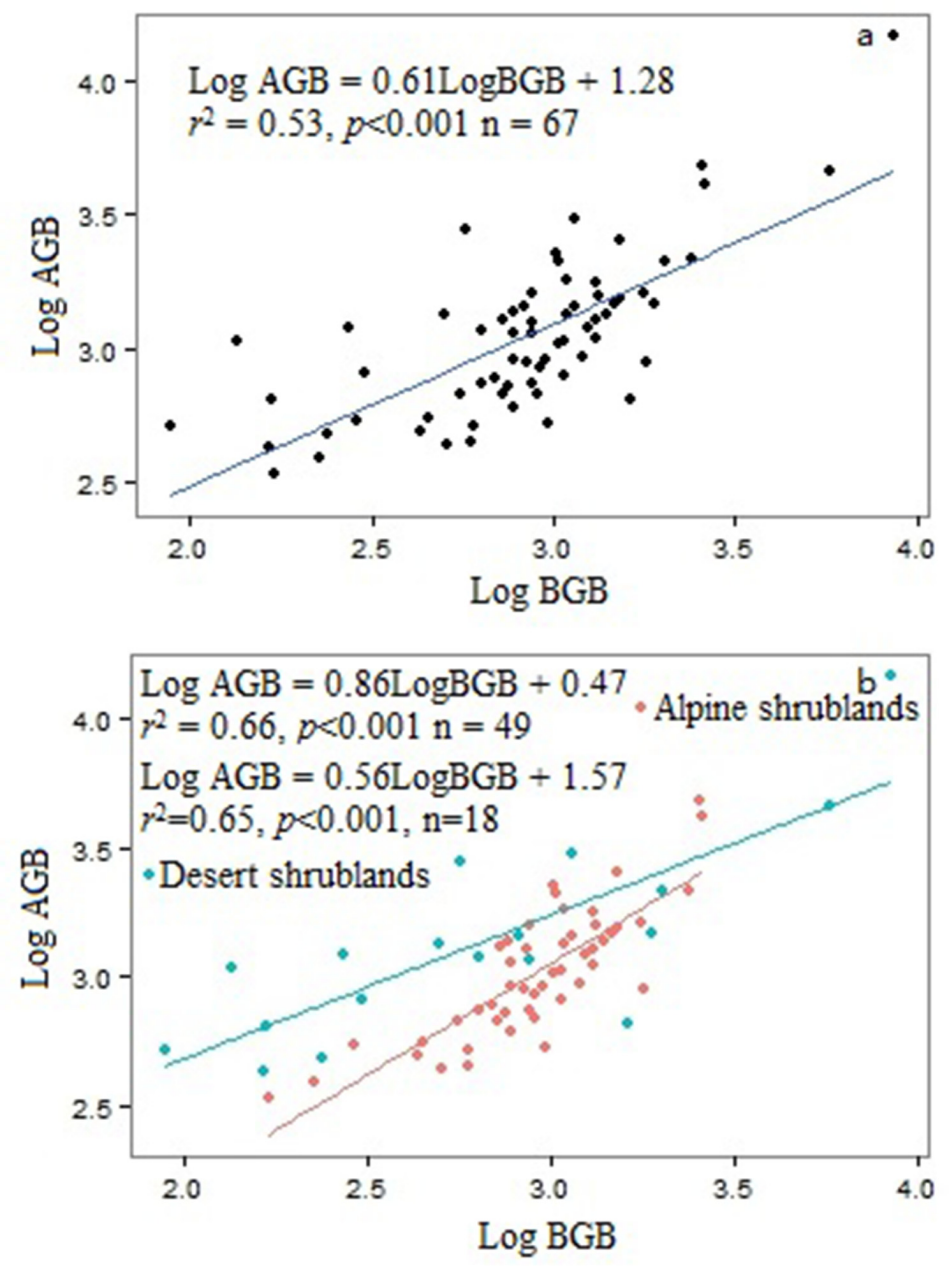
Relationships between above-ground biomass (AGB) and below-ground biomass (BGB) in Tibetan Plateau shrubs. (a): the slope of the relationship between log AGB and log BGB for overall shrub was 0.61, with 95% confidence intervals of 0.46–0.75. (b): the red line denotes the allocation relationship for alpine shrubs, while the green line indicates the relationship for desert shrubs. The 95% confidence intervals of the slopes for alpine shrubs and desert shrubs were 0.68–1.04 and 0.34–0.78, respectively.

The slope (a) of the allometric relationship between log AGB and log BGB for overall shrubs was 0.61, with 95% confidence interval of 0.46–0.75 (Fig 3A), which was significant different from the isometric relationships. Furthermore, AGB in desert shrubs scales with BGB in a different manner to alpine shrubs. The allocation of biomass in the alpine shrubs was supported by isometric hypothesis while the relationship in desert shrubs was supported by allometric allocation hypothesis (Fig 3B).

## Discussion

### Size of R/S

The median R/S is 1.84 in global shrubs [4], which is larger than that in shrub biomes across the northeast Tibetan Plateau. Furthermore, the average R/S is 1.2 in global sclerophyllous shrubs [5], which is also higher than desert shrub and alpine shrubs on the Tibetan Plateau. What is responsible for this difference? From a physiological perspective, R/S has been interpreted as reflecting the different investment of photosynthates between aboveground and belowground organs [3]. Then, compared with R/S ratios in global regions, shrubs in Tibetan Plateau allocate more biomass to AGB. It has been proven that the growing season of plant is shorted [34] and the CO_2_ partial pressure markedly decreases [35,36] with the increasing altitude, which may undermine the maximum photosynthetic rate [37]. As plants generally allocate biomass to the organs that acquire the most limiting resource [8]. Thus, the plant growing at high altitude may attend to invest more biomass to AGB for obtaining higher photosynthetic rate to adapt on the Tibetan Plateau. To explore whether grasslands have the same characteristic, we compared R/S on the Tibetan Plateau with that on the Inner Mongolia, China. We found that R/S of grasslands on the Tibetan Plateau is 5.8 [9] and smaller than that on the Inner Mongolia, which is 6.3 [38]. These phenomena demonstrate that the botany on the Tibetan Plateau may tend to allocate more photosynthates to aboveground organs to fit the maximizing growing capacity.

### Relationships between climatic factors on biomass partitioning

The R/S in Tibetan shrubs dropped significantly with increasing in MAT (*r*^*2*^ = 0.08, *p* < 0.05 Fig 2A). This deduction was similar to the significant decreasing trend of R/S with increasing temperature [2,4] and global grasslands, but different from the trend of China’s grasslands [14]. The higher R/S in regions might be slower root turnover in colder regions [39], and also could be associated with the relatively slow depletion of carbohydrates in roots, resulting from low respiration rates in cold regions [40]. Consequently, R/S may be significantly negative correlation with MAT. The R/S in our study showed a significantly positive trend with MAP (*r*^*2*^ = 0.12, *p* < 0.05 Fig 2B). This result was opposite to the relationships in global shrubs and grasslands [4] and different that R/S in China’s grasslands did not show any significant trend with MAP [14]. In northeast Tibetan Plateau shrubs, MAP was negatively corrected with MAT (*r* = -0.4, *p* < 0.05). Therefore, with increasing precipitation, temperature would be lower, which may lead to increase of R/S. Furthermore, different water use efficiency might be derived from the differences in vegetational and biogeochemical constraints [41,42], which may lead to a more efficiency use of precipitation in BGB across Tibetan Plateau shrubs.

### Allometric relationships between AGB and BGB

The allocation of biomass in alpine shrubs is supported by isometric hypothesis while it is supported by allometric allocation hypothesis in desert shrubs. In the harsh ecosystems, scarce precipitation allows plants to allocate more biomass to the aboveground organs, which is favour for plants to survive [13,17]. Therefore, biomass allocation in the desert shrub and alpine shrubs may support the different allocation hypothesis. Similarly, Wu et al. (2013) reported that in the more rough Tibetan Plateau alpine steppe ecosystem, the relationship between BGB and AGB supports the allometric biomass partitioning hypothesis [17], while biomass allocation in the alpine grasslands reflects the isometric allocation hypothesis [9].

Considering the aboveground biomass and belowground biomass in Tibetan Plateau grasslands allocation is supported by isometric hypothesis [9]. Different biomass allocation equations should be considered when estimating belowground biomass from aboveground at different biomes. Though the generality of isometric relationship exists between log AGB and log BGB at the level of woody and non-woody communities [9], we further demonstrated that different biomass allocation hypothesis for particular biomes. Similarly, across the 20 different forest- level regressions in China’s forest, which are not statistically distinguishable from the isometric relationship [31], but not all forests fit to isometric relationship, such as temperate mixed coniferous-broadleaf forest, temperate deciduous broadleaf forest [31]. Therefore, the ‘universal’ exponents do not exist for all biomes and in order to improve the accuracy of root biomass estimates, specific allocation equations should be used for given biomes.

## Conclusion

This study is the first to document information on biomass allocation and the relationships between R/S and climatic factors in shrub biomes on the Tibetan Plateau. We found that R/S was sensitive to MAP and MAT. The AGB in Tibetan Plateau shrub did not scale strikingly with BGB and the slop was significantly different from 1.0. The result supported the optimal relationship and it was similar to desert shrub. Interestingly, the slop of alpine shrub was significantly supporting the isometric relationship. These findings suggest that different biomes allocation equations should be considered when estimating BGB from AGB for differ shrub biomes.

## Supporting Information

S1 Table Description of 67 sites in shrub biomes across the northeast Tibetan Plateau. Data for latitude, longitude, AGB and BGB.
(PDF)
