# Supplementary material for: Above- and Belowground Biomass Allocation in Shrub Biomes across the Northeast Tibetan Plateau
Source: PLoS One. 2016 Apr 27;11(4):e0154251. doi: 10.1371/journal.pone.0154251 (PMC4847786; doi:10.1371/journal.pone.0154251)
Supplement: S1 Table — Data for latitude, longitude, AGB and BGB. (PDF) [file pone.0154251.s001.pdf]

S1 Table. Description of 67 sites in shrub biomes across the northeast Tibetan Plateau. Data for latitude, longitude, AGB and BGB.

latitude	longitude	AGB(g/m^2)	BGB(g/m^2)
36.72	102.38	740.76	636.28
37.04	98.66	883.24	843.11
36.90	98.52	1447.77	825.02
36.99	98.20	426.03	164.37
37.05	97.63	14623.12	8565.27
36.19	94.78	1334.97	498.07
36.45	98.39	1078.69	135.75
36.49	96.25	4579.84	5781.15
37.40	101.99	1776.79	1304.79
37.24	101.85	551.67	452.17
34.45	100.21	1617.57	1776.22
32.90	100.92	942.47	1208.75
32.89	100.66	1527.27	1532.24
33.38	101.34	1813.00	1086.90
33.37	101.35	1060.12	1069.74
33.27	100.63	897.13	1814.80
33.97	99.90	1263.08	869.35
33.85	99.84	391.95	227.91
34.51	100.08	524.44	967.00
35.42	102.24	802.62	1063.90
35.41	102.23	493.13	433.37
35.53	102.25	4816.52	2558.41
35.41	102.25	2270.79	1023.44
35.23	101.85	676.49	722.32
36.32	101.93	674.28	559.71
37.38	101.41	723.89	754.31
38.09	100.35	609.24	775.45
38.06	100.24	1439.85	1149.85
33.04	96.86	1569.51	1329.20
33.03	96.84	1203.58	1249.24
33.05	96.85	916.86	782.19
33.04	96.85	848.11	912.99
31.88	96.58	1339.04	1084.20
31.88	96.89	1595.98	874.91
32.89	95.30	1134.81	778.68
33.33	97.12	743.42	872.85
33.32	97.12	1102.55	1306.93
33.36	96.99	2554.55	1534.03
37.13	99.52	2098.98	1039.98
37.43	100.26	1286.50	1303.02

37.43	100.26	685.57	908.29
37.17	99.16	4142.78	2597.28
37.53	99.32	437.53	506.95
37.53	99.33	2159.59	2379.54
37.37	99.33	1375.96	771.19
36.86	98.57	1468.57	1895.71
36.87	98.54	1146.20	869.90
36.96	98.86	340.34	170.21
37.35	97.12	817.36	303.32
37.45	97.26	541.94	289.70
37.04	97.86	2140.37	2039.23
37.26	97.41	646.98	167.52
37.22	97.51	2819.14	569.63
35.88	94.53	518.21	89.36
35.88	94.51	1207.57	273.01
36.00	97.41	480.90	238.70
35.88	94.55	3025.76	1140.71
36.41	98.67	654.41	1639.54
36.39	98.73	1181.14	637.08
36.37	101.57	771.94	693.98
36.28	102.24	1297.66	732.00
36.27	101.96	912.09	951.55
37.00	102.13	1473.69	1478.39
37.29	101.43	1036.36	1026.95
35.85	99.92	451.55	595.24
35.47	100.74	514.71	599.77
36.38	101.55	1353.17	1406.66
